# Conserved Organisation of 45S rDNA Sites and rDNA Gene Copy Number among Major Clades of Early Land Plants

**DOI:** 10.1371/journal.pone.0162544

**Published:** 2016-09-13

**Authors:** Marcela Rosato, Aleš Kovařík, Ricardo Garilleti, Josep A. Rosselló

**Affiliations:** 1 Jardín Botánico, ICBiBE-Unidad Asociada CSIC, Universidad de Valencia, c/Quart 80, E-46008, Valencia, Spain; 2 Institute of Biophysics, Academy of Sciences of the Czech Republic, Brno, CZ–61265, Czech Republic; 3 Departamento de Botánica, Facultad de Farmacia, Universidad de Valencia, E-46100, Burjassot, Spain; 4 Marimurtra Bot. Garden, Carl Faust Fdn., PO Box 112, E-17300, Blanes, Catalonia, Spain; Agriculture and Agri-Food Canada, CANADA

## Abstract

Genes encoding ribosomal RNA (rDNA) are universal key constituents of eukaryotic genomes, and the nuclear genome harbours hundreds to several thousand copies of each species. Knowledge about the number of rDNA loci and gene copy number provides information for comparative studies of organismal and molecular evolution at various phylogenetic levels. With the exception of seed plants, the range of 45S rDNA locus (encoding 18S, 5.8S and 26S rRNA) and gene copy number variation within key evolutionary plant groups is largely unknown. This is especially true for the three earliest land plant lineages Marchantiophyta (liverworts), Bryophyta (mosses), and Anthocerotophyta (hornworts). In this work, we report the extent of rDNA variation in early land plants, assessing the number of 45S rDNA loci and gene copy number in 106 species and 25 species, respectively, of mosses, liverworts and hornworts. Unexpectedly, the results show a narrow range of ribosomal locus variation (one or two 45S rDNA loci) and gene copies not present in vascular plant lineages, where a wide spectrum is recorded. Mutation analysis of whole genomic reads showed higher (3-fold) intragenomic heterogeneity of *Marchantia polymorpha* (Marchantiophyta) rDNA compared to *Physcomitrella patens* (Bryophyta) and two angiosperms (*Arabidopsis thaliana* and *Nicotiana tomentosifomis*) suggesting the presence of rDNA pseudogenes in its genome. No association between phylogenetic position, taxonomic adscription and the number of rDNA loci and gene copy number was found. Our results suggest a likely evolutionary rDNA stasis during land colonisation and diversification across 480 myr of bryophyte evolution. We hypothesise that strong selection forces may be acting against ribosomal gene locus amplification. Despite showing a predominant haploid phase and infrequent meiosis, overall rDNA homogeneity is not severely compromised in bryophytes.

## Introduction

Genes encoding ribosomal RNA (rDNA) are universal key constituents of eukaryotic genomes because their products form the backbones of the functional cytoplasmic, plastid, and mitochondrial ribosomes [1]. In contrast with the single or low-copy number of rRNA genes present in the plastidial and mitochondrial genomes, the nuclear genome harbours hundreds to several thousand copies of each ribosomal species (18S, 5.8S, 25S/26S, 5S) that are usually arranged in distinct arrays of tandemly-repeated cistrons (45S, formed by the 18S, 5.8S, 25S/26S units) or genes (5S) [2].

Nuclear rDNA has long been regarded as being merely involved in the biogenesis of ribosomes and the nucleolus [3–5], but recent evidence has dramatically changed this perception, suggesting that it is also involved in other cell processes. Thus, it has been hypothesised that the rDNA constitutes a central factor in the maintenance and organisation of the genome, modulating cellular homeostasis by acting to preserve genome stability, triggering cell aging and senescence, and regulating genome damage resistance [6, 7], maintaining genome-wide chromatin structure [8], and modulating variation in gene expression across the genome [9]. It has also been suggested that rDNA variation in gene copy number has a significant impact on the evolutionary ecology of all organisms, mediated through increased phosphorus demand in organisms with high rRNA content [10].

Although rDNA is one of the most abundant gene families occupying a large fraction of the nuclear genome, it is also structurally one of the most unstable genomic regions [6]. The reasons for this instability are not fully understood; however it has been reported that rDNA loci are the predominant sites of repeated recombination [11]. Thus, unequal recombination between homologous and homoelogous loci may trigger both intragenomic fluctuation in rDNA copy number and amplification of new arrays [12, 13]. Furthermore, it has been shown that rDNA arrays and neighbouring regions are one of the frequent targets for mobile element insertions [14]. Transposition may promote the evolutionary dynamics of rDNA loci not only across species radiation but also during intraspecies differentiation and domestication, producing karyological rearrangements that may be at the ongoing of speciation processes, towards genetic differentiation [15].

Knowledge about the number of rDNA loci, their genomic location, and rDNA linkage provides information for comparative studies of organismal and molecular evolution at various phylogenetic levels. With the exception of seed plants, where a large body of data has been accumulated about the number of ribosomal loci by means of molecular cytogenetic techniques [16], the range of 45S rDNA locus and gene copy variation within key evolutionary plant groups is largely unknown. This is especially true for the three earliest land plant lineages Marchantiophyta (liverworts), Bryophyta (mosses), and Anthocerotophyta (hornworts), where data for less than five species representing only two systematic families have been gathered [17–19].

Knowledge about structural chromosomal features in early embryophytes dates back to the early 20th century [20]. However, the aims of the cytogenetic research, other than assessing chromosomal number, have mainly focused on the presence of sex and accessory chromosomes, types and patterns of heterochromatin distribution, and the nature of micro-chromosomes ([20–22] and references therein). The study of fine chromosome morphology in bryophytes is a difficult task due to their low mitotic index, the usually small chromosome size and structural uniformity, and uncertainties inherent in establishing the centromere position in metaphase chromosomes [23, 24]. It is not surprising that the recognition of nucleolar organising regions (NOR) has not been a priority task for most of the studies researching bryophyte cytogenetics [21]. Moreover, some studies noted the difficulty of adequately interpreting the primary or secondary constrictions in telocentric bryophyte chromosomes using conventional cytological techniques based on orcein stains [25], due to terminal centromeres or very shorts arms of acrocentric chromosomes being wrongly recognised as satellites (e.g., [26]). Thus, reports on NOR number in early embryophytes were based on the attachment of specific chromosomes to nucleoli [27], but no staining methods specific for the detection of active NOR (silver impregnation or staining) [28] have been used to verify these observations.

**Fig 1:**
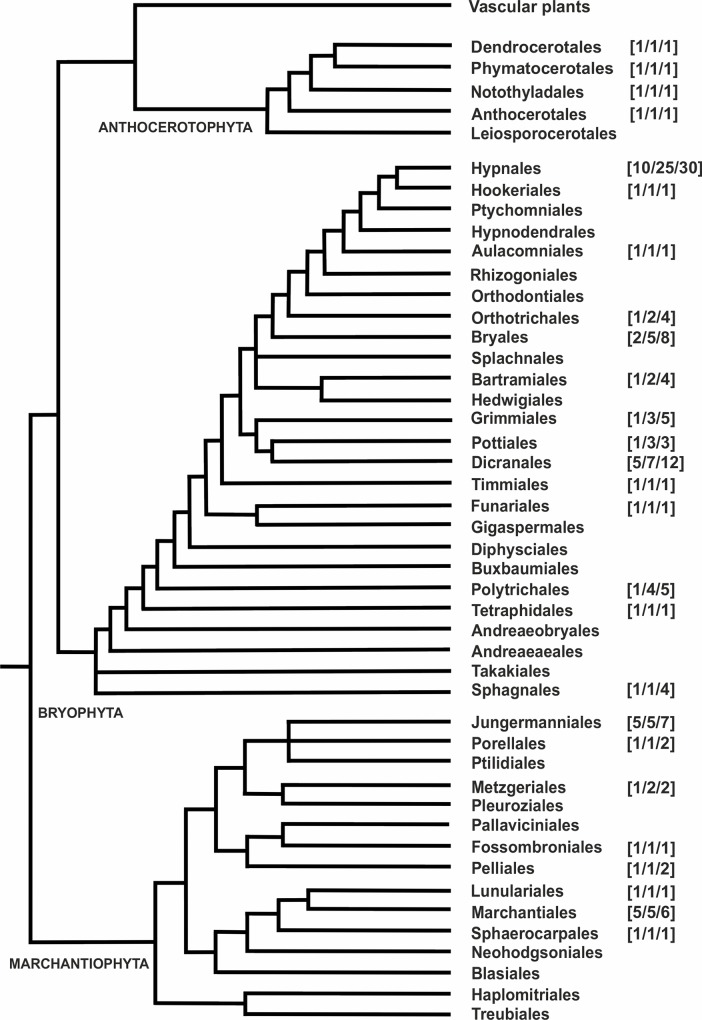
Consensus backbone phylogeny of the higher clades (orders) of the three early land plant groups, liverworts (Marchantiophyta), mosses (Bryophyta), and hornworts (Anthocerotophyta), based on molecular phylogenetic data. Tree based on Hilger [29]. Numbers on the right indicate the families, genera, and species sampled from each taxonomic group.

In this work, we assess the extent of rDNA variation in the number of 45S rDNA loci and gene copy number in early land plants, sampling the most species high-order clades (Fig 1). The main aims were (i) to explore to what extent land plant colonisation was linked to drastic genome reorganisations in NOR number, (ii) to assess whether nuclear DNA content are related to the number of 45S rDNA loci and gene copy number, and (iii) to compare the dynamics of rDNA site and gene amplification in three groups of land plants showing a predominant haploid life cycle phase with that reported for the predominant diploid vascular plants.

## Materials and Methods

### Plant material

Living specimens belonging to 106 species were collected from Japan, Costa Rica, Portugal, Continental Spain, Balearic Islands, and Slovenia (Fig 1, S1 Table), placed in polyethylene bags and transported to the lab at the Botanical Garden of Valencia University. Part of the material was dried, or fixed in an ethanol/glacial acetic acid mixture (3:1) for thallose liverworts and hornworts and kept as a voucher specimen (VAL). The remainder was placed in a polythene bag and grown in the lab under dark conditions at room temperature or eventually at 4°C until used. The species *Urginea undulata* (2n = 20; one locus equals two sites), *Ginkgo biloba* (2n = 24; two loci), *Urginea maritima* (2n = 60; three loci), and *Vella pseudocytisus* subsp. *glabrata* (2n = 34; four loci), were used as control plants to assess the correspondence between counting the number of FISH sites from metaphase chromosomes and from interphase nuclei. The field studies did not involve any endangered or protected species and no specific permissions were required for sampling.

### Cytogenetic analysis

Living young apices were excised from gametophytes or, more rarely, from the sporophytic tissue (hornworts), fixed in an ethanol/glacial acetic acid mixture (3:1) and eventually stored at –20°C until required. For somatic nuclei preparations, the gametophyte apices were washed in 10 mM citrate buffer (pH 4.6) and then macerated in a mixture of 4% (v/v) cellulase (Calbiochem, Darmstadt, Germany) in 10 mM citrate buffer (pH 4.6) and 20% pectinase (from *Aspergillus niger*) in 40% glycerol for 90 min at 37°C. For thallose liverworts and hornworts, the concentration of pectinase was doubled (40%) [19]. The spreading procedure of Zhong et al. [30] was followed to prepare nuclei for *in situ* hybridisation.

#### Ag-NOR staining

Silver impregnation was carried out on 1–2 day-old preparations according to the protocol described in Rosato and Rosselló [31]. Slides were incubated with a few drops of freshly prepared 50% silver nitrate solution and covered with a nylon mesh, and incubated in a humid chamber at 55–60°C for 1–2 h. After washing in distilled water, slides were stained with 4% Giemsa solution for 5 min., quickly rinsed in distilled water, air-dried, and mounted in 50% glycerol solution.

#### Fluorescence *in situ* hybridisation (FISH)

The 45S rDNA multigene family was localised using the pTa71 [32] clones. The pTa71 probe was labelled with digoxigenin-11dUTP using the Nick translation kit (Roche, Germany). We followed the *in situ* hybridisation protocols of Rosato et al. [33], except for the proteinase K pre-treatment, which was performed following Schwarzacher and Heslop-Harrison [34]. The hybridisation mix contained 2 μg mL-^1^ of 45S rDNA labelled probe in 50% formamide, 2 x SSC, 0.25% SDS (sodium dodecyl sulphate) and 10% dextran sulphate. Hybridisation was carried out at 37°C during 12–16 h, after which washes were performed in 1 × SSC for 30 min at 37°C. Probe detection was conducted using the method of Zhong et al. [30]. The first detection step was carried out with anti-digoxigenin-fluorescein (15 μg mL^-1^, Roche, Germany) in 4B buffer [0.5% BSA (bovine serum albumin) in 4 x SSC, 0.05% (v/v) Tween-20] and the second step with fluorescein conjugate rabbit-anti-mouse (1:100, Merck, UK) in TNB (0.5% BSA in 100 mM Tris-HCL pH 7.5, 150 mM NaCl).

Hybridisation signals were analysed using an epifluorescence Olympus microscope equipped with an Olympus Camedia C-2000-Z digital camera, with appropriate filters set. The images were optimised for contrast and brightness by image processing software (Adobe Photoshop v. 7.0).

#### Quantitative PCR

qPCR was used to estimate haploid gene copies by comparing the rate of amplification of samples to that of *Arabidopsis thaliana* cv. Landsberg erecta for which the rDNA copy number per genome haploid (1C) is known (570/1C) [35]. The amplicon comprised 220 bp of the 26S gene 3’end [36]. The primers were as follows: 26S_for: 5’-GAATTCACCCAAGTGTTGGGAT-3’; 26S_rev: 5’-AGAGGCGTTCAGTCATAATC-3’. The 20 μl PCR reaction mixture contained the primers annealing to the conserved part of the 26S gene. Sequences of primers were checked in the assembled *Physcomitrella patens* and *Marchantia polymorpha* units (further below). The 20 μl PCR mix contained 1 μl (10 pmol) of each primer, 10 μl of 2 x SYBR Green mix (Roche), 5 μl of genomic DNA (5 ng) and 3 μl of distilled water. The cycling conditions were as follows: initial denaturation at 94°C for 7 min followed by 40 cycles of 20 s at 94°C, 20 s at 57°C and 30 s at 72°C; and a final extension step at 72°C, 10 min). The SYBR Green I fluorescence was monitored consecutively after the extension step. The quality of products was checked by thermal denaturation cycle. Only results providing a single peak (78–81) were considered. Generally, the cycle threshold (ct) values ranged between 13 and 16 when setting used a baseline of ~10^4^ fluorescence units. The copies in an unknown DNA sample were calculated according to equation: 2^[ct unknown sample–ct reference]^ x 570 x [1C of sample/1C of *A*. *thaliana*], where the reference is *A*. *thaliana* DNA, *1C* is the genome size in Mb and *570* is the number of copies in *A*. *thaliana*. Each analysis was carried out in three technical replicates (using the same template DNA but different reactions), and the values were averaged. Particular attention was paid to DNA concentration estimates employing two independent methods: (i) SYBR Green-based fluorimetric assay according to the protocol implemented within the Corbett Rotor-Gene RG-3000A thermocycler program, (ii) comparison of fluorescence signals after the electrophoresis of DNAs in agarose gels using a series dilution of lambda DNA standards. An actin 1 gene (GenBank AY3822282.1) from *P*. *patens* was used as an internal control of amplification efficiency. The primers for the actin 1 gene (5’-CACCACACGTTCTACAAC-3’ and 5’-CCCTTCTCCCATCACTCA-3’) amplified a 209 bp product. The conditions for amplification were similar as described above with few exceptions: higher amounts of template (25 ng) were used and the annealing temperature was 50°C. The ct values ranged 31–34 indicating some degradation and/or copy number variation. Severely degraded DNAs were excluded from the analysis (S1 Table).

#### Analysis of rDNA intragenomic homogeneity and gene copies from NGS reads

The 45S (18S-ITS1-5.8S-ITS2-26S) were assembled from NGS Illumina reads of *Marchantia polymorpha* (SRR1800537), *Physcomitrella patens* (SRR1685734) and *Nicotiana tomentosiformis* (SRR343065). The reference guided assembly involved the following steps: (i) Reference sequence selection. Sequences of clones in Genbank (AY342318, AY342317, JN089185, X74114.1, X51576.1, AJ012362 and EU161982) were used as references. The clones contained either the full length 18S-ITS1-5.8S-ITS2-26S unit (*M*. *polymorpha*) or its subregions (*P*. *patens*, *A*. *thaliana* and *N*. *tomentosiformis*). To obtain full length units from the fragmented information, subregions were aligned and assembled using the BioEdit program. (ii) Quality and length trimming. The starting read pool of NGS reads consisted of more than ten of millions of unpaired reads. Before the mapping (‘MAP READ REFERENCE’ tool) all reads with Ns, reads less than 90 nt in length or reads failing to pass a quality scores limit of p = 0.05 were removed using a ‘TRIM’ command in the CLCbio. The mapping parameters were as follows: mismatch cost value 2, insertion cost value 3, deletion cost value 3, with both the length fraction value and the similarity fraction value set at 0.8. The read coverage of rDNA exceeded 100 x in most cases with some variation in the ITS regions. (iii) Extraction of consensus sequences. The consensus sequences were obtained from mapped reads (‘EXTRACT CONSENSUS SEQUENCE’) and checked for the correct order in the BioEdit program.

The number of 26S gene copies were calculated as follows: (i) Genome proportion (GP) was calculated from the number of mapped reads to the 26S reference divided by the total reads in percentages. (ii) Calculation of GP in Mb: GP x size of the genome in Mb. (iii) Copies were then calculated: GP values in Mb divided by the size of a 26S gene (~0.003 Mb).

Variations were then detected via the `Probabilistic Variant Detection´ function tool in CLCbio, using default settings. SNPs were filtered as follows: Minimum read coverage– 100, Count (the number of countable reads supporting the allele)—10, frequency (the ratio of “the number of 'countable' reads supporting the allele” to “the number of 'countable' reads covering the position of the variant”): ≥ 10% (high frequency SNPs); deletions and insertions were removed from data sets. The SNP data in a tabular format were exported to the MsExcel program for further statistics and graphical representation.

## Results

### rDNA loci analysed by molecular cytogenetics

The disposition of 45S rDNA sites in interphase nuclei has a similar organisation to that previously known in plants and patterns of rDNA chromatin organisation associated with the nucleolus corroborate an observation and classification in vascular plants [37]. Condensed rDNA chromatin in interphase nuclei are adjacent to the nucleolus (perinucleolar knob) and the rDNA sites detected by FISH co-localise with heterochromatin knobs at interphase. The presence of knobs corresponding to rDNA chromatin (knob-rDNA chromatin like) was restricted to one or, on rare occasions, two FISH 45 rDNA sites.

**Fig 2:**
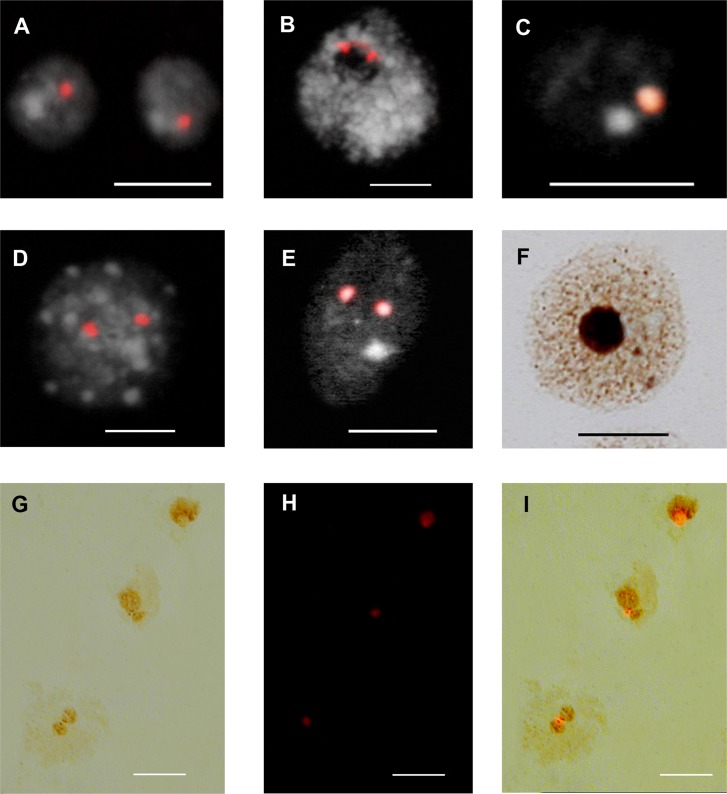
Patterns of rDNA organization, 45S rDNA loci number, and nucleolar activity in species of Marchantiophyta, Bryophyta, and Anthocerotophyta (liverworts, mosses and hornworts, respectively). A-E, FISH of rRNA genes using the 45S rDNA probe. F Ag-NOR staining. G-I, Sequential Ag-NOR and FISH. A, *Thuidium delicatulum*. B, *Pellia epiphylla* and C, *Neckera crispa* showing a single site signal. D, *Pellia endiviifolia* and E, *Campylopus introflexus*, showing two sites. F, Ag-NOR staining in interphase nuclei of *Pellia epiphylla* showing one large nucleolus. G-H, Sequential Ag-NOR (G) and FISH (H) in *Atrichum undulatum*. The Ag-staining in several interphase nuclei shows one nucleolus (top right), two homomorphic (left bottom), and two heteromorphic nucleoli (middle). I, merged G and H images. Scale bars: 10 μm.

With the exception of two species all other accessions showed a single FISH signal (Table 1; S1 Table; Fig 2A, 2B and 2C), irrespective of the nuclei stage during cell development, suggesting that there might be a single locus based on a single knob-rDNA chromatin like signal located around the nucleolus. In *Pellia epiphylla* the two knob signals (Fig 2B) were interconnected by a diffuse string-like signal indicating largely decondensed and presumably active chromatin interspersed between two heterochromatic blocks of inactive genes. The species *Pellia endiviifolia* and *Campylopus introflexus* showed the presence of two sites (Fig 2D and 2E). In these two species, Ag-NOR staining of interphase nuclei showed that the two rDNA sites were active and contributed to the nucleolus formation.

**Table 1:** Number of rDNA FISH signals in interphase nuclei and gene copy number estimates for liverwort (Marchantiophyta) and moss (Bryophyta) species jointly analysed (excepting *Physcomitrella patens*). **Genome size values were taken from Bennet and Leitch [38].** The number of rDNA copies were determined by qPCR or calculated from whole genomic sequences (indicated by an asterisk). nd: not determined.

	Number of FISH signals	Number of rDNA copies/1C	Genome size (pg/1C)
**Marchantiophyta**			
**Marchantiales**			
*Marchantia polymorpha* L. subsp. *polymorpha*	1	1263 ± 126	0.29–0.59
		2003*	
*Conocephalum conicum* (L.) Underw.	1	670 ± 161	0.69
**Lunulariales**			
*Lunularia cruciata* (L.) Dumort. ex Lindb.	1	693 ± 118	0.67
**Pelliales**			
*Pellia endiviifolia* (Dicks.) Dumort.	2	1970 ± 315	3.44
**Porellales**			
*Porella platyphylla* (L.) Pfeiff.	1	1169 ± 245	1.09
**Jungermanniales**			
*Plagiochila porelloides* (Nees) Lindberg.	1	2003 ± 361	1.57
*Bazzania trilobata* (L.) Gray	1	666 ± 73	0.95
**Bryophyta**			
**Polytrichales**			
*Atrichum undulatum* (Hedw.) P. Beauv.	1	1169 ± 129	0.73
*Polytrichum piliferum* Hedw.	1	2458 ± 467	0.48
**Funariales**			
*Encalypta streptocarpa* Hedw.	1	1897 ± 304	0.45
*Physcomitrella patens* (Hedw.) Bruch & Schimp.	nd	903 ± 45	0.53
		653*	
**Dicranales**			
*Fissidens dubius* P. Beauv.	1	1238 ± 173	0.36
**Pottiales**			
*Syntrichia ruralis* (Hedw.) F. Weber & D. Mohr	1	1595 ± 175	0.39
*Tortella tortuosa* (Hedw.) Limpr.	1	704 ± 85	0.33
**Bryales**			
*Bryum capillare* Hedw.	1	667 ± 167	0.59
*Plagiomnium undulatum* (Hedw.) T.J. Kop.	1	2070 ± 248	0.47
**Aulacomniales**			
*Aulacomnium palustre* (Hedw.) Schwägr.	1	735 ± 147	0.32
**Hypnales**			
*Isothecium alopecuroides* (Lam. ex Dubois)	1	659 ± 86	0.48
*Homalothecium lutescens* (Hedw.) H. Rob.	1	1678 ± 252	0.48
*Hylocomium splendens* (Hedw.) Schimp.	1	1007 ± 171	0.39
*Hypnum cupressiforme* Hedw.	1	1359 ± 285	0.41
*Neckera complanata* (Hedw.) Huebener	1	858 ± 206	0.45
*Neckera crispa* Hedw.	1	678 ± 142	0.50
*Thamnobryum alopecurum* (Hedw.) Gangulee	1	696 ± 139	0.47
*Thuidium delicatulum* (Hedw.) Schimp.	1	434 ± 87	0.30–0.39

In a few species showing a single 45S rDNA locus, two nucleoli of similar or different size were observed. In these accessions, sequential Ag-NOR staining and FISH revealed that the single 45S rDNA hybridisation signal was associated with both nucleoli (Fig 2G–2I).

### Gene copies determined by quantitative PCR and *in silico* analysis of whole genomic reads

To determine gene copies we carried out qPCR analysis using the 26S gene-specific primers. The sample collection contained 25 species, belonging to Marchantiophyta and Bryophyta lineages plus the *Arabidopsis thaliana* and *Nicotiana tomentosiformis* controls, for which rDNA copies are known (570 and 1900, respectively) [35, 36]. The rDNA copy numbers ranged from about 500 to 2500 (Table 1). Median was 1007 and average 1197 ± 630. To confirm the qPCR results, selected samples were analysed by slot-blot hybridisation (S2 Fig). The intensity of hybridisation signals was comparable between the species indicating little variation.

The gene copies in *Physcomitrella patens*, *Funaria hygrometrica* and *Marchantia polymorpha* were also calculated from the number of mapped reads extracted from whole genome sequence archives (Table 2).

**Table 2:** Copy number of rDNA in genomes calculated from NGS reads.

	Genomesize^1^(Mb)	Sequence read archive	Total reads	Mapped reads(26S)	GP(%)	GP(Mb)	26Scopies
*Physcomitrella patens*	480	SRR191864	131650374	528177	0.40	1.93	~650^2^
*Marchantia polymorpha*	287	SRR1800537	141348695	3486319	2.47	7.08	~2400
*Funaria hygrometrica*	394	n.a.^3^			3.51^4^	13.8	~1200
*Nicotiana tomentosiformis*	2500	SRR343065	7479035	19115	0.26	6.39	~1800

^1^The values were taken from Bennet and Leitch [38].

^2^Analysis of other two sequence archives, SRR400524 and SRR0722296, resulted in estimates of 650 and 750 copies, respectively.

^3^Sequence archive is not available. The GP was taken from Liu et al. [39].

^4^ GP is calculated for the whole 11.2 kb unit.

The values calculated for *P*. *patens* were highly congruent with those experimentally determined. There were no significant differences between three different Illumina runs. Similarly, the copies for *N*. *tomentosiformis* were in good agreement with those previously published [36]. In *M*. *polymorpha*, the calculated copies from NGS reads were about 2 fold larger than those of qPCR-determined.

### Structure of rDNA units and inter species comparisons

The sizes of assembled 18S-ITS1-5.8S-ITS2-26S units (without the IGS) were 5311, 6937 and 5824 bp in *P*. *patens*, *M*. *polymorpha* and *N*. *tomentosiformis*, respectively. Another moss species, *F*. *hygometrica*, had a unit length of 5538 bp [39]. The pairwise sequence divergences are shown in S2 Table. The divergences ranged 0.07–0.18 roughly reflecting phylogenetic relationships. Most variation was located in the ITS1 and ITS2 subregions. There were few length polymorphisms in the coding regions caused mainly by short (1–113 bp long) insertion/deletion events (indels). Both ITS1 and ITS2 subregions displayed considerable inter species length variability: *M*. *polymorpha* had the longest ITS1 and ITS2 corresponding to 1150 and 2011 bp, respectively; *P*. *patens* had ITS1 and ITS2 sizes of 308 and 325 bp, respectively, which are similar to the lengths seen in most angiosperms. The ITS2 of *P*. *patens* had exceptionally high sequence coverage among the NGS reads (>10 fold increase compared to other subregions) which may indicate occurrence of ITS2-related sequences outsides of the rDNA unit.

### Intragenomic rDNA homogeneity determined from whole genomic reads

The intragenomic homogeneity of multicopy gene families is a significant parameter reflecting the mode of their evolution. In order to determine the intragenomic homogeneity we analysed polymorphisms along the 18S-ITS1-5.8S-ITS2-26S region in sequenced genomes of two bryophyte (*Marchantia polymorpha* and *Physcomitrella patens*) and two angiosperm (*Arabidopsis thaliana* and *Nicotiana tomentosiformis*) species.

**Fig 3:**
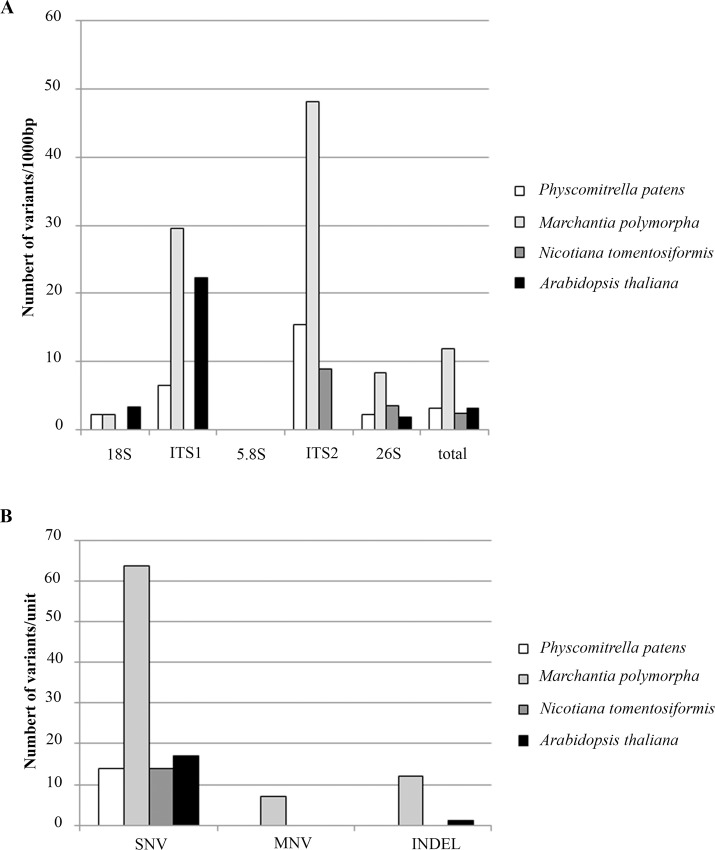
Intragenomic homogeneity of 45S rDNA. A, Distribution of variants along the 18S-ITS1-5.8S-ITS2-26S units. Mutation analysis was carried out in four species using the mapped Illumina reads. Position of mutations and their frequencies are given in S3 Table. B, Type of individual polymorphisms within the rDNA unit: SNV (single nucleotide variation), MNV (multiple nucleotide variation, 2 or more consecutive mutations). Indel (sum of insertions and deletions).

For a mutation analysis at genomic scales, we considered the high frequency variation occurring at ≥ 10% frequency, i.e. about 100 genes would carry such a variant (considering there may be ~1000 copies in the genome). Quantification of three kinds of sequence variation (single nucleotide variation, multiple nucleotide variation and indels) along the unit is shown in Fig 3A (for source data, see S3 Table). As expected, most SNPs accumulated in both ITSs which are under weaker selection constrains than the coding regions. Out of the four species analysed, *M*. *polymorpha* had the far largest intragenomic diversity of rDNA. The frequencies of individual variant types are shown in Fig 3B. Again, *M*. *polymorpha* was exceptional in having relatively high frequency of longer (≥2 nucleotides) polymorphisms (multiple nucleotide variation) and the indels.

## Discussion

The analysis of 106 species belonging to the three basal land plant lineages (Marchantiophyta, 22 species; Bryophyta, 80 species, Anthocerophyta, 4 species) includes accessions from 79 genera, 48 families, and 26 orders. In addition, 17 species were represented by at least two accessions. Thus, our study provides a reasonable sample size from a wide phylogenetic coverage to assess the patterns of rDNA variation in early embryophytes.

We are aware of the limited or lack of biological replicates for most species in our sampling. However, we believe that the basic information concerning the conserved organisation of the 45S rDNA sites and the range of rRNA gene copy number would not be hampered by the analysis of more samples. However, population level-study in selected species might be interesting to address the question of what level of rDNA sequence variability is present within populations. Although it is sometimes high among the angiosperms owing to the presence of frequent multiloci rDNA genotypes, we believe could be low in bryophytes from the reasons discussed below.

Cytogenetic work is not an easy task in bryophytes due to the recognised low mitotic index present in these organisms. With our pre-treatment approach, and despite noticeable technical effort, we have not been able to score metaphase chromosomes in the analysed accessions. Hence, the cytogenetic analysis has been restricted to analyse FISH signals in interphase nuclei in as many cells as possible. Admittedly, FISH signals may not be an accurate way to calculate number of chromosome sites as the one-dimensional interphase nucleus cannot fully show the total number of 45S rDNA sites due to potential overlaps that may underestimate the total number of loci present. For these reasons, we have several control plant species for which the number of chromosome sites from metaphase rDNA FISH was available, and for which corresponding interphase nuclei images were scored (S1 Fig). This resulted in congruent results between both counting approaches. In addition, a previous study indicated a good correlation between the number of metaphase and interphase rDNA signals in genus *Pellia* [19]. Together, it seems that the assessment of the number of sites from interphase FISH signals is feasible in bryophyte systems.

### The number of ribosomal loci is conserved across early embryophyte lineages

All species analysed in this study show a narrow range of ribosomal locus variation (one or two 45S rDNA loci) that are equivalent to that previously reported in three liverworts from two genera (*Marchantia*, 2 loci, [17]; *Pellia* 1–2 loci, [19]) using FISH. In addition, these values completely agree with the presence in liverworts and moss species of 1 or 2 NOR chromosomes, and 1 or 2 nucleoli as assessed by conventional staining [20, 27, 40]. Orzechowska and colleagues [19] analysed *Pellia epiphylla* from a different population. Yet, both studies are fully congruent in that there is a single rDNA site in this species. On the other hand, the closely related *Pellia endiviifolia* showed two loci indicating some variation at the species level, as previously reported by Newton [41] who noted one or two NOR throughout the species range. Of note, *Pellia* had almost tenfold higher DNA amount than the rest of the bryophytes (Table 1) indicating variation in genome size possibly linked to polyploidy and/or expansion of heterochromatin [19].

Our results in *Marchantia polymorpha* are congruent with those reported by Sone et al. [17] except of a minute additional rDNA site detected in the previous study. This small divergence may be explained by technical reasons due to the use of homologous [17] or heterologous (this work) FISH probes. By contrast, Nakayama et al. [42] and Fujisawa et al. [43] reported nine and ten rDNA sites (in male and female individuals, respectively). The striking differences reported by these authors and the results obtained by Sone et al. [17] and ourselves requires alternative explanations. Nakayama et al. [42] and Fujisawa et al. [43] used a 0.5 kb ribosomal probe in their FISH experiments. This might have detected not only canonical rDNA units but also the presence of (partially) amplified non-functional 18S rRNA genes that could lead to false interpretations as rDNA sites. In addition, cell cultures, as those used by Nakayama et al. [42] and Fujisawa et al. [43], are usually unstable and may exist as a community of cells with different karyotypes reflected in different chromosome numbers, morphologies and distributions of satellite repeats and rDNA variants [44].

### Chromosome number and the number of 45S rDNA loci and copies are not associated in early land plants

The basal chromosome number for each major lineage of early plants is not known with certainty, and the topic has been much debated albeit with tenuous arguments at best [20–22, 45, 46]. The fact that two active NOR loci were reported in *Riccardia pinguis* [27] led Berrie [40] to speculate that liverworts with n = 10 were polyploids from n = 5 ancestors belonging to Anthocerotophyta. He further suggested that liverworts with n = 9, the basic chromosome number virtually present in all families and genera of hepatics [46] except for *Riccia* and allies [47], were also polyploids that evolved from n = 10 ancestors.

The hypothesis of Berrie [40] was based on the misconception that the presence of two nucleolar chromosomes in the haploid complement of a plant is as a sign of polyploidy. Phylogenetic studies using molecular markers from the three genomes did not support Berrie [40] suggestions. In fact, multiple lines of evidence suggest that the three bryophyte clades form a paraphyletic basal grade, supporting the hypothesis that liverworts were the most ancestral diverging lineage of land plants, whereas hornworts evolved after the moss divergence and are believed to be the sister group of vascular plants ([48–50], but see [51] for an alternative hypothesis).

Reported chromosome numbers for the analysed species in this work spanned a wide range of variation, from n = 6 in *Philonotis* sp. pl. to n = 42 in *Sphagnum* species [52]. There seems to be no association between chromosome numbers and 45S rDNA sites and copies appears in our sampling, and it is unlikely that this trend would change when additional species are analysed. In fact, the presence of one or two ribosomal loci has been found in the congeneric homoploid (n = 9) *Pellia epiphylla* and *P*. *endiviifolia* species, respectively.

### Why is there an evolutionary stasis in the number of 45S rDNA loci and gene copy across early embryophytes lineages?

The assessment of the number of 45S rDNA loci and copies in major clades of early land plants has revealed an unexpected narrow range of variation (1–2 loci) not present in other vascular plant lineages, where a wide spectrum is recorded [53–55]. Our results, based on a wide sampling across taxonomic and evolutionary boundaries, suggest a likely evolutionary rDNA stasis during land colonisation and diversification across 480 myr of bryophyte evolution.

This pattern is intriguing as the 45S rDNA cistrons are reputed to be one of the most dynamic multigene families in plant genomes, even over very short evolutionary time [15, 56]. Theoretically, the increase in the number of 45S rDNA loci could be explained by several genomic processes that are potentially acting on distantly related eukaryotic organisms. Thus, ectopic recombination between non-homologous chromosomes may cause inter-chromosomal interchange of rDNA gene copies [57]. There is compelling evidence that interlocus exchange driving homogenisation of rDNA across chromosomes [58, 59] is rare in some bryophytes: (i) Previous study reported amplification of a chromosome specific IGS variants in *Marchantia polymorpha*. (ii) Here we observed reduced intragenomic homogeneity of 45S genes in this species suggesting the presence of pseudogenes. This pattern was not so obvious in *P*. *patens* which had an intragenomic homogeneity (high) comparable to that of both angiosperm species, *A*. *thaliana* and *N*. *tomentosiformis*. Thus, observations made on a single species cannot be generalised. The ITS diversity in angiosperms is often attributed to the presence of multiple loci in the genome and/or frequent inter species hybridisation events ([60]; and for review see [61]). None of these mechanisms seems to be applicable in bryophytes where hybridisation is infrequent, and which typically harbour only a single locus (Table 1). Perhaps, the ITS diversity could be explained by infrequent meiotic recombination since bryophytes spend most of their life cycle in the haploid stage. Nevertheless, coding parts of rDNA units were relatively homogeneous (as in most angiosperms) indicating strong selection pressures imposed on their functionality. The degree of pseudogenisation is therefore lower than in some gymnosperms in which most of the genes were assigned to pseudogenes [62].

Alternatively, the origin of new 45S rDNA sites could be linked to the activation of mobile elements which can produce a transposition of rDNA copies to new genomic locations [63]. The intragenomic mobility of rRNA genes as a consequence of transposon activity has been widely reported in seed plants, and it has been hypothesised that it is one of the major forces driving rDNA locus evolution (e.g., [64]). Because there is no empirical evidence supporting the view that these mechanisms of potential rDNA amplification are absent or not operating in early land plants we hypothesise that strong selection forces may be acting against ribosomal gene locus amplification.

Several key life-cycle features of liverworts, mosses and hornworts might be associated with selection pressures against increasing the number of rDNA loci and thus associated to this rDNA stasis. These groups harbour a conserved linked arrangement of 5S and 45S genes [17, 65, 66, 67] suggesting that the rDNA stasis may also manifest at the unit level.

First, all these organisms are characterised by having a predominant (in space and time) gametophytic haploid generation in contrast with the fugacious and gametophyte-dependent diploid sporophytic phase [68]. This means that, in contrast to diploid or polyploid free-living generations, where at least one pair of homologous chromosomes is present, genetic buffering against non-optimal or deleterious mutations is not possible. It remains unexplained how the apparent high level of homologous recombination reported recently in *Physcomitrella patens* is realised in a gametophyte phase of organism growth [69].

Second, different studies have assessed that chromosome breakpoints are located near or within chromosomal segments mainly composed of heterochromatin [70]. In particular, the plant 45S rDNA regions are fragile sites prone to chromosomal lesions [71]. Expanding the number of 45S rDNA loci above a critical threshold would also increase the likelihood of chromosome breakage in ribosomal DNA regions and, accordingly, genome rearrangements of uncertain fitness in predominant haploid individuals.

Third, mosses, hornworts and, to a lesser extent, liverworts usually show low average 1C genome size values and a narrow range of variation between extreme values that are at the lower end of those reported for land plants [72–75]. Similarly to the genome size, rRNA gene copies exhibit a narrow (five fold variation) range compared to seed plants that exhibit much larger (sixty fold) variation [76]. Also the average number of gene copies relatively small (~1200) among bryophytes while angiosperms typically harbour several thousands of copies. These observations fit with the observed correlation between genome size and number of rRNA genes [77]. Certainly, ferns considered to be early land plants possess large genomes while they show slow evolution as bryophytes and mosses [78]. It will be interesting to determine number of rDNA loci and their intragenomic variation in these organisms.

## Conclusions

In this work, we demonstrated rDNA locus and copy number stasis in bryophytes. Together with their relatively intragenomic sequence heterogeneity it is likely that expansion/homogenisation cycles are relatively rather infrequent in this group. Although the results of the analysis of a single locus do not allow us to extrapolate these findings to the whole genome it is tempting to speculate that expansion of repeated sequences are under strong selection constraints in bryophytes explaining, thus, to their relatively small and uniform genome sizes. It is an open question whether these features are connected with the gametophyte dominant life cycle. However, it has been suggested that in archegoniate plants producing biflagellated sperm gametes (such as early land plants), lower DNA contents afford a selective advantage through a nucleotypic effect on sperm cell efficiency and effectiveness, which in turn influences reproductive success [79].

## Supporting Information

S1 Fig **Physical mapping of rDNA loci in interphase nuclei (A-D) and metaphase chromosomes (E-H) of control plant species.** A,E, *Urginea undulata* (one locus). B,F, *Ginkgo biloba* (two loci). C,G, *Urginea maritima* (three loci). D,H, *Vella pseudocytisus* subsp. *glabrata* (four loci). Dots in F refer to a decondensed site. Scale bars: 10 μm. (TIFF 1.25 Mb)
(TIF)

S2 Fig rDNA amounts in the genomes analysed by slot- blot hybridisation. Series dilutions of genomic DNA and a 220 bp PCR product amplified from the 26S rRNA gene of *P*. *patents* were hybridised with the 26S probe of *P*. *patens* (probe was the same PCR product). (TIFF 197 Kb)
(TIF)

S1 Table Accessions of Marchantiophyta, Bryophyta, and Anthocerotophyta analysed by FISH. The number of 45S rDNA loci and the published haploid chromosome number for each species (when available) are reported. (DOC 195 kb)
(DOC)

S2 Table Nucleotide divergences between the 45S sequences (coding region plus the internal transcribed spacers). The number is given as the Jukes-Kantor distance between identical and overlapping alignment positions between the two sequences. (XLSX 24 Kb)
(XLSX)

S3 Table Summary of mutation analysis of 45S genes in *Physcomitrella patens*, *Marchantia polymorpha*, *Arabidopsis thaliana* and *Nicotiana tomentosiformis*. Information about the position, type of polymorphism, variant count, nucleotide coverage and frequency is given in the columns. Three types of polymorphisms were scored: single nucleotide variation (SNV), multiple nucleotide variation (MNV) and indels (sum of deletions and insertions). The polymorphisms in the coding regions are highlighted in yellow. (XLSX 33 Kb)
(XLSX)
