# Supplementary figures and images for: Conserved Organisation of 45S rDNA Sites and rDNA Gene Copy Number among Major Clades of Early Land Plants
Source: PLoS One. 2016 Sep 13;11(9):e0162544. doi: 10.1371/journal.pone.0162544 (PMC5021289; doi:10.1371/journal.pone.0162544)

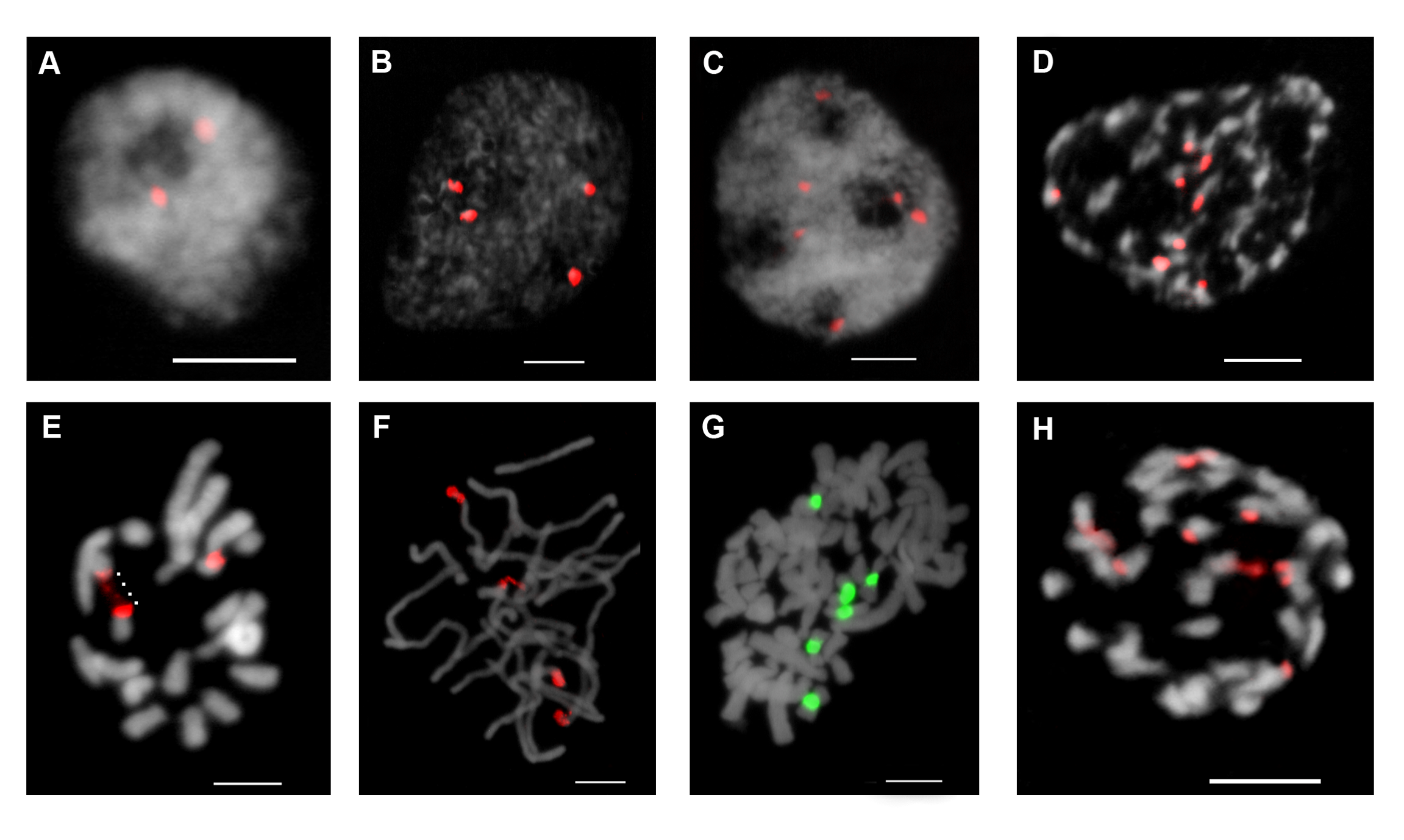

Supplement: S1 Fig — Physical mapping of rDNA loci in interphase nuclei (A-D) and metaphase chromosomes (E-H) of control plant species. A,E, Urginea undulata (one locus). B,F, Ginkgo biloba (two loci). C,G, Urginea maritima (three loci). D,H, Vella pseudocytisus subsp. glabrata (four loci). Dots in F refer to a decondensed site. Scale bars: 10 μm. (TIFF 1.25 Mb) (TIF) [file pone.0162544.s001.tif]

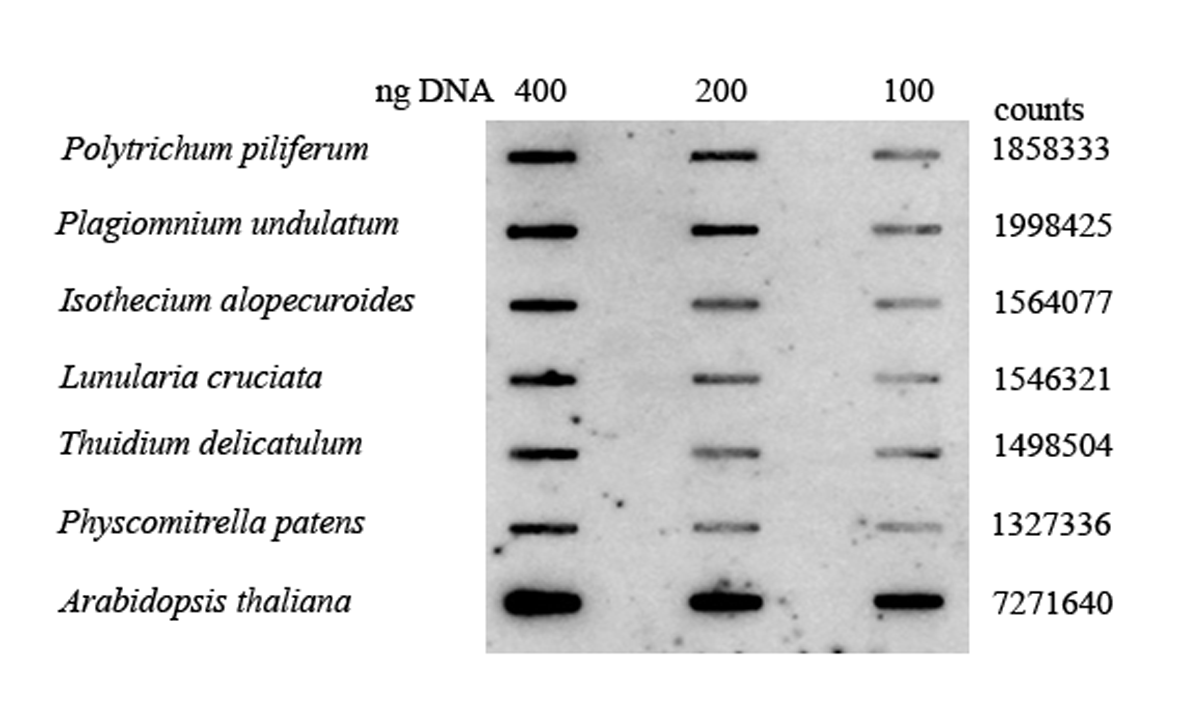

Supplement: S2 Fig — Series dilutions of genomic DNA and a 220 bp PCR product amplified from the 26S rRNA gene of P. patents were hybridised with the 26S probe of P. patens (probe was the same PCR product). (TIFF 197 Kb) (TIF) [file pone.0162544.s002.tif]
