# Supplementary material for: Conserved Organisation of 45S rDNA Sites and rDNA Gene Copy Number among Major Clades of Early Land Plants
Source: PLoS One. 2016 Sep 13;11(9):e0162544. doi: 10.1371/journal.pone.0162544 (PMC5021289; doi:10.1371/journal.pone.0162544)
Supplement: S1 Table — The number of 45S rDNA loci and the published haploid chromosome number for each species (when available) are reported. (DOC 195 kb) (DOC) [file pone.0162544.s003.doc]

**Additional file 1: Table S1.** Accessions of Marchantiophyta, Bryophyta, and Anthocerotophyta analyzed by FISH. The number of 45S rDNA loci and the published haploid chromosome number for each species (when available) are reported.

| Species | Phyllum, Order, Family | Origin | No. of 45S loci | Chromosome number |
| --- | --- | --- | --- | --- |
| *Amphidium mougeotii* (Schimp.) Schimp. | Bryophyta, Dicranales, Rhabdoweisiaceae | Spain, Cuenca, Valdemeca, Arroyo Chico, 26-8-2012, J.A. Rosselló | 1 | - |
| *Anthoceros caucasicus* Stephani | Anthocerotophyta, Anthocerotales, Anthocerotaceae | Portugal, Sintra, 18-6-2013, C. Garcia | 1 | - |
| *Antitrichia curtipendula* (Hedw.) Brid. | Bryophyta, Hypnales, Leucodontaceae | Spain, Cuenca, Valdemeca, Arroyo Chico, 26-8-2012, J.A. Rosselló | 1 | - |
| *Antitrichia curtipendula* (Hedw.) Brid. | Bryophyta, Hypnales, Leucodontaceae | Spain, Cuenca, Valdemeca, 20-5-2012, J.A. Rosselló | 1 | - |
| *Apometzgeria pubescens* (Schrank) Kuwah. | Marchantiophyta, Metzgeriales, Metzgeriaceae | Spain, Lleida, between Benabé and Pla de Beret, 24-7-2012, J.A. Rosselló | 1 | 8, 9 |
| *Atrichum undulatum* (Hedw.) P. Beauv. | Bryophyta, Polytrichales, Polytrichaceae | Spain, Lleida, Vall Ferrera, 25-7-2012, J.A. Rosselló | 1 | 7, 14, 21 |
| *Atrichum undulatum* (Hedw.) P. Beauv. | Bryophyta, Polytrichales, Polytrichaceae | Spain, Lleida, between Benabé and Pla de Beret, 24-7-2012, J.A. Rosselló | 1 | 7, 14, 21 |
| *Aulacomnium palustre* (Hedw.) Schwägr. | Bryophyta, Rhizogoniales, Aulocomniaceae | Spain, Cuenca, Valdemeca, 20-5-2012, J.A. Rosselló | 1 | 12 |
| *Bartramia halleriana* Hedw. | Bryophyta, Bartramiales, Bartramiaceae | Spain, Lleida, between Benabé and Pla de Beret, 24-7-2012, J.A. Rosselló | 1 | 9, 12 |
| *Bartramia pomiformis* Hedw. | Bryophyta, Bartramiales, Bartramiaceae | Spain, Cuenca, Valdemeca, Arroyo Chico, 26-8-2012, J.A. Rosselló | 1 | 8, 9 |
| *Bazzania trilobata* (L.) Gray | Marchantiophyta, Jungermanniales, Lepidoziaceae | Slovenia: Bohinj lake, 23-8-2012, J. Miravet | 1 | 9, 10 |
| *Brachytheciastrum dieckei* (Röll) Ignatov & Huttunen | Bryophyta, Hypnales, Brachytheciaceae | Spain, Cuenca, Valtablado, 25-8-2012, J.A. Rosselló | 1 | - |
| *Bryum capillare* Hedw. | Bryophyta, Bryales, Bryaceae | Spain, Cuenca, Valdemeca, Arroyo Chico, 26-8-2012, J.A. Rosselló | 1 | 10, 12, 20 |
| *Bryum pseudotriquetrum* (Hedw.) P. Gaertn., B. Mey. & Scherb. | Bryophyta, Bryales, Bryaceae | Spain, Cuenca, Valdemeca, Arroyo Chico, 26-8-2012, J.A. Rosselló | 1 | 10, 11 |
| *Calliergonella cuspidata* (Hedw.) Loeske | Bryophyta, Hypnales, Hypnaceae | Spain, Cuenca, Valdemeca, Arroyo Chico, 26-8-2012, J.A. Rosselló | 1 | 11 |
| *Calliergonella cuspidata* (Hedw.) Loeske | Bryophyta, Hypnales, Hypnaceae | Spain, Cuenca, Valtablado, 25-8-2012, J.A. Rosselló | 1* | 11 |
| *Campyliadelphus elodes* (Lindb.) Kanda | Bryophyta, Hypnales, Hypnaceae | Spain, Cuenca, Valtablado, 25-8-2012, J.A. Rosselló | 1 | - |
| *Campylopus introflexus* (Hedw.) Brid. | Bryophyta, Dicranales, Leucobryaceae | Spain, Barcelona, Tordera, 30-7-2010, M.Jover | 2 | - |
| *Chiloscyphus polyanthos* (L.) Corda | Marchantiophyta, Jungermanniales, Lophocoleaceae | Spain, Cuenca, Valdemeca, Arroyo Chico, 26-8-2012, J.A. Rosselló | 1 | 9 |
| *Climacium dendroides* (Hedw.) F. Weber & D. Mohr | Bryophyta, Hypnales, Climaciaceae | Spain, Lleida, Vall Ferrera, Pla de Boet, 25-7-2012, J.A. Rosselló | 1 | 11 |
| *Conocephalum conicum* (L.) Underw. | Marchantiophyta, Marchantiales, Conocephalaceae | Spain, Castelló, Eslida, Font Matilde, 27-8-2012, J. Miravet | 1 | 9 |
| *Conocephalum salebrosum* Szweykowski, Buczkowska & Odrzykoski | Marchantiophyta, Marchantiales, Conocephalaceae | Spain, Lleida, between Benabé and Pla de Beret, 24-7-2012, J.A. Rosselló | 1 | - |
| *Conocephalum salebrosum* Szweykowski, Buczkowska & Odrzykoski | Marchantiophyta, Marchantiales, Conocephalaceae | Slovenia: Bohinj lake, 23-8-2012, J. Miravet | 1 | - |
| *Ctenidium molluscum* (Hedw.) Mitt. | Bryophyta, Hypnales, Hylocomiaceae | Slovenia: Bohinj lake, 23-8-2012, J. Miravet | 1 | 8 |
| *Dichodontium palustre* (Dicks.) M.Stech | Bryophyta, Dicranales, Rhabdoweisiaceae | Spain, Lleida, between Tabascan and Noarre, 23-7-2012, J.A. Rosselló | 1 | 15 |
| *Dicranum crassifolium* Sérgio, Ochyra & Séneca | Bryophyta, Dicranales, Dicranaceae | Slovenia: Bohinj lake, 23-8-2012, J. Miravet | 1 | - |
| *Dicranum scoparium* Hedw. | Bryophyta, Dicranales, Dicranaceae | Spain, Cuenca, Valdemeca, 20-5-2012, J.A. Rosselló | 1* | 11, 12, 13, 14, 17 |
| *Dicranum tauricum* Sapjegin | Bryophyta, Dicranales, Dicranaceae | Spain, Lleida, between Benabé and Pla de Beret, 24-7-2012, J.A. Rosselló | 1 | 12, 14 |
| *Ditrichum gracile* (Mitt.) Kuntze | Bryophyta, Dicranales, Ditrichaceae | Spain, Lleida, between Benabé and Pla de Beret, 24-7-2012, J.A. Rosselló | 1 | 13 |
| *Ditrichum heteromallum* (Hedw.) E. Lawton | Bryophyta, Dicranales, Ditrichaceae | Spain, Cuenca, Valdemeca, Arroyo Chico, 26-8-2012, J.A. Rosselló | 1 | 13, 14 |
| *Drepanocladus aduncus* (Hedw.) Warnst. | Bryophyta, Hypnales, Amblistegiaceae | Spain, Lleida, Vall Ferrera, 25-7-2012, J.A. Rosselló | 1 | 12 |
| *Drepanocladus aduncus* (Hedw.) Warsnt. | Bryophyta, Hypnales, Amblistegiaceae | Spain, Cuenca, Valtablado, 25-8-2012, J.A. Rosselló | 1 | 12 |
| *Dumortiera hirsuta* (Sw.) Nees | Marchantiophyta, Marchantiales, Dumortieraceae | Japan, Kyushu, Yakushima Is., Shiratani Unsuikkyo, 18-9-2012, R. Garilleti & F. Lara | 1 | 9, 18, 27 |
| *Dumortiera hirsuta* (Sw.) Nees | Marchantiophyta, Marchantiales, Dumortieraceae | Japan, Kyushu, Yakushima Is., Shiratani Unsuikkyo, 18-9-2012, R. Garilleti & F. Lara | 1 | 9, 18, 27 |
| *Encalypta streptocarpa* Hedw. | Bryophyta, Funariales, Encalyptaceae | Spain, Cuenca, Valtablado, 25-8-2012, J.A. Rosselló | 1 | 13 |
| *Fissidens dubius* P. Beauv. | Bryophyta, Dicranales, Fissidentaceae | Spain, Cuenca, Valdemeca, Arroyo Chico, 26-8-2012, J.A. Rosselló | 1 | 12. 13, 15, 16 |
| *Fissidens grandifrons* Brid. | Bryophyta, Dicranales, Fissidentaceae | Spain, Lleida, between Benabé and Pla de Beret, 24-7-2012, J.A. Rosselló | 1 | 10, 12 |
| *Fossombronia* sp. | Marchantiophyta, Fossombroniales, Fossombroniaceae | Balearic Islands, Minorca, Maó, 21-2-2012, J.A. Rosselló | 1 | - |
| *Grimmia torquata* Hornsch. ex Drumm. | Bryophyta, Grimmiales, Grimmiaceae | Spain, Cuenca, Valdemeca, Arroyo Chico, 26-8-2012, J.A. Rosselló | 1 | - |
| *Grimmia trichophylla* Grev. | Bryophyta, Grimmiales, Grimmiaceae | Spain, Cuenca, Valdemeca, Arroyo Chico, 26-8-2012, J.A. Rosselló | 1 | 13, 26 |
| *Herzogiella seligeri* (Brid.) Z. Iwats. | Bryophyta, Hypnales, Hypnaceae | Spain, Lleida, between Benabé and Pla de Beret, 24-7-2012, J.A. Rosselló | 1 | 11 |
| *Homalothecium aureum* (Lag.) H. Rob. | Bryophyta, Hypnales, Brachytheciaceae | Spain, Cuenca, Valdemeca, Arroyo Chico, 26-8-2012, J.A. Rosselló | 1 | - |
| *Homalothecium lutescens* (Hedw.) H. Rob. | Bryophyta, Hypnales, Brachytheciaceae | Spain, Cuenca, Valtablado, 25-8-2012, J.A. Rosselló |  | 8, 10, 11, 12, 14 |
| *Homalothecium lutescens* (Hedw.) H. Rob. | Bryophyta, Hypnales, Brachytheciaceae | Spain, Cuenca, Valdemeca, Arroyo Chico, 26-8-2012, J.A. Rosselló | 1 | 8, 10, 11, 12, 14 |
| *Hookeria lucens* (Hedw.) Sm. | Bryophyta, Hookeriales, Hookeriaceae | Spain, Cuenca, Valdemeca, 20-5-2012, J.A. Rosselló | 1 | 12 |
| *Hylocomium splendens* (Hedw.) Schimp. | Bryophyta, Hypnales, Hylocomiaceae | Spain, Cuenca, Valdemeca, 20-5-2012, J.A. Rosselló | 1 | 10, 11, 12 |
| *Hypnum andoi* A.J.E. Sm. | Bryophyta, Hypnales, Hypnaceae | Slovenia: Bohinj lake, 23-8-2012, J. Miravet | 1 | 10 |
| *Hypnum cupressiforme* Hedw. | Bryophyta, Hypnales, Hypnaceae | Slovenia: Bohinj lake, 23-8-2012, J. Miravet | 1 | 10, 11 |
| *Isothecium alopecuroides* (Lam. ex Dubois) Isov. | Bryophyta, Hypnales, Lembophyllaceae | Spain, Cuenca, Valdemeca, Arroyo Chico, 26-8-2012, J.A. Rosselló | 1 | 10, 11, 12 |
| *Isothecium alopecuroides* (Lam. ex Dubois) Isov. | Bryophyta, Hypnales, Lembophyllaceae | Spain, Cuenca, Valdemeca, Arroyo Chico, 26-8-2012, J.A. Rosselló | 1 | 10, 11, 12 |
| *Isothecium alopecuroides* (Lam. ex Dubois) Isov. | Bryophyta, Hypnales, Lembophyllaceae | Slovenia: Bohinj lake, 23-8-2012, J. Miravet | 1 | 10, 11, 12 |
| *Lejeunea cavifolia* (Ehrh.) Lindb. | Marchantiophyta, Jungermanniales, Lejeuneaceae | Spain, Cuenca, Valdemeca, Arroyo Chico, 26-8-2012, J.A. Rosselló | 1 | 9 |
| *Leptodictyum riparium* (Hedw.) Warnst. | Bryophyta, Hypnales, Amblistegiaceae | Spain, Cuenca, Valtablado, 25-8-2012, J.A. Rosselló | 1 | 10, 12, 20, 21, 24, 40, 46 |
| *Leucobryum juniperoideum* (Brid.) Müll. Hal. | Bryophyta, Dicranales, Leucobryaceae | Slovenia: Bohinj lake, 23-8-2012, J. Miravet | 1 | - |
| *Lunularia cruciata* (L.) Dumort. ex Lindb. | Marchantiophyta, Lunulariales, Lunulariaceae | Spain, Castelló, Eslida, Font Matilde, 27-8-2012, J. Miravet | 1 | 9 |
| *Marchantia polymorpha* L. subsp*. polymorpha* | Marchantiophyta, Marchantiales, Marchantiaceae | Spain, León, Campa de Mortera, 2-9-2012, F. del Egido | 1 | 9 |
| *Metzgeria conjugata* Lindb. | Marchantiophyta, Metzgeriales, Metzgeriaceae | Slovenia: Bohinj lake, 23-8-2012, J. Miravet | 1* | 18 |
| *Mnium stellare* Hedw. | Bryophyta, Bryales, Mniaceae | Spain, Lleida, between Tabascan and Noarre, 23-7-2012, J.A. Rosselló | 1 | 7 |
| *Neckera complanata* (Hedw.) Huebener | Bryophyta, Hypnales, Neckeraceae | Spain, Lleida, between Tabascan and Noarre, 23-7-2012, J.A. Rosselló | 1 | - |
| *Neckera crispa* Hedw. | Bryophyta, Hypnales, Neckeraceae | Spain, Lleida, between Benabé and Pla de Beret, 24-7-2012, J.A. Rosselló | 1 | 11 |
| *Neckera crispa* Hedw. | Bryophyta, Hypnales, Neckeraceae | Slovenia: Bohinj lake, 23-8-2012, J. Miravet | 1 | 11 |
| *Neckera menziesii* Drumm. | Bryophyta, Hypnales, Neckeraceae | Spain, Cuenca, Valdemeca, Arroyo Chico, 26-8-2012, J.A. Rosselló | 1 | - |
| *Orthotrichum anomalum* Hedw. | Bryophyta, Orthotrichales, Orthotrichaceae | Spain, Cuenca, Valtablado, 25-8-2012, J.A. Rosselló | 1 | 11 |
| *Orthotrichum cupulatum* Hoffm. | Bryophyta, Orthotrichales, Orthotrichaceae | Spain, Cuenca, Valdemeca, Arroyo Chico, 26-8-2012, J.A. Rosselló | 1 | 11 |
| *Orthotrichum sp.* | Bryophyta, Orthotrichales, Orthotrichaceae | Spain, Cuenca, Valtablado, 25-8-2012, J.A. Rosselló | 1 | - |
| *Oxyrrhynchium schleicheri* (R. Hedw.) Röll | Bryophyta, Hypnales, Brachytheciaceae | Slovenia: Bohinj lake, 23-8-2012, J. Miravet | 1 | - |
| cf. *Oxystegus* sp. | Bryophyta, Pottiales, Pottiaceae | Slovenia: Bohinj lake, 23-8-2012, J. Miravet | 1 | - |
| *Palustriella commutata* (Hedw.) Ochyra var. *commutata* | Bryophyta, Hypnales, Amblistegiaceae | Spain, Cuenca, Valdemeca, 26-8-2012, J.A. Rosselló | 1 | 10 |
| *Pellia endiviifolia* (Dicks.) Dumort. | Marchantiophyta, Pelliales, Pelliaceae | Spain, Lleida, between Benabé and Pla de Beret, 24-7-2012, J.A. Rosselló | 2 | 9 |
| *Pellia endiviifolia* (Dicks.) Dumort. | Marchantiophyta, Pelliales, Pelliaceae | Spain, Castelló, Eslida, 27-8-2012, J. Miravet | 2 | 9 |
| *Pellia epiphylla* (L.) Corda | Marchantiophyta, Pelliales, Pelliaceae | Spain, Lleida, Vall Ferrera, 25-7-2012, J.A. Rosselló | 1* | 9 |
| *Pellia epiphylla* (L.) Corda | Marchantiophyta, Pelliales, Pelliaceae | Spain, Lleida, Vall Ferrera, 25-7-2012, J.A. Rosselló | 1 | 9 |
| *Pellia epiphylla* (L.) Corda | Marchantiophyta, Pelliales, Pelliaceae | Spain, Lleida, Vall Ferrera, 25-7-2012, J.A. Rosselló | 1 | 9 |
| *Pellia epiphylla* (L.) Corda | Marchantiophyta, Pelliales, Pelliaceae | Spain, Lleida, Vall Ferrera, 25-7-2012, J.A. Rosselló | 1 | 9 |
| *Pellia epiphylla* (L.) Corda | Marchantiophyta, Pelliales, Pelliaceae | Spain, Lleida, between Benabé and Pla de Beret, 24-7-2012, J.A. Rosselló | 1 | 9 |
| *Pellia epiphylla* (L.) Corda | Marchantiophyta, Pelliales, Pelliaceae | Spain, Cuenca, Valdemeca, Arroyo Chico, 26-8-2012, J.A. Rosselló | 1 | 9 |
| *Phaeomegaceros* sp. nov. | Anthocerotophyta, Dendrocerotales, Dendrocerotaceae | Costa Rica, Volcán Irazu, 11-7-2006, J.C. Villarreal, N. Wickett & Dauphin 871 | 1 | - |
| *Phaeoceros laevis* (L.) Prosk. | Anthocerotophyta, Notothyladales, Notothyladaceae | Portugal, Sintra, Botanical Garden, 18-6-2013, C. Garcia | 1 | - |
| *Philonotis fontana* (Hedw.) Brid. | Bryophyta, Bartramiales, Bartramiaceae | Spain, Lleida, between Tabascan and Noarre, 23-7-2012, J.A. Rosselló | 1 | 6 |
| *Philonotis tomentella* Molendo | Bryophyta, Bartramiales, Bartramiaceae | Spain, Lleida, Vall Ferrera, 25-7-2012, J.A. Rosselló | 1 | 6 |
| *Phymatoceros bulbiculosus* (Brot.) Stotler, W.T. Doyle & Crand.-Stot. | Anthocerotophyta, Phymatocerotales, Phytomatocerotaceae | Portugal, Sintra, 18-6-2013, C. Garcia | 1 | - |
| *Plagiochila asplenioides* (L.) Dumort. | Marchantiophyta, Jungermanniales, Plagiochilaceae | Spain, Lleida, between Benabé and Pla de Beret, 24-7-2012, J.A. Rosselló | 1 | 9 |
| *Plagiochila porelloides* (Nees) Lindberg. | Marchantiophyta, Jungermanniales, Plagiochilaceae | Slovenia: Bohinj lake, 23-8-2012, J. Miravet | 1 | 9 |
| *Plagiochila porelloides* (Torr. ex Nees) Lindenb. | Marchantiophyta, Jungermanniales, Plagiochilaceae | Spain, Cuenca, Valdemeca, 20-5-2012, J.A. Rosselló | 1 | 9 |
| *Plagiomnium elatum* (Bruch & Schimp.) T.J.Kop. | Bryophyta, Bryales, Miniaceae | Spain, Cuenca, Valdemeca, 20-5-2012, J.A. Rosselló | 1 | 6, 12 |
| *Plagiomnium elatum* (Bruch & Schimp.) T.J.Kop. | Bryophyta, Bryales, Miniaceae | Spain, Cuenca, Valdemeca, Arroyo Chico, 26-8-2012, J.A. Rosselló | 1 | 6, 12 |
| *Plagiomnium medium* (Bruch & Schimp.) T.J.Kop. | Bryophyta, Bryales, Miniaceae | Spain, Lleida, Vall Ferrera, Pla de Boet, 25-7-2012, J.A. Rosselló | 1 | 6, 12, 13 |
| *Plagiomnium undulatum* (Hedw.) T.J. Kop. | Bryophyta, Bryales, Miniaceae | Spain, Lleida, between Benabé and Pla de Beret, 24-7-2012, J.A. Rosselló | 1 | 6, 7 |
| *Plagiomnium undulatum* (Hedw.) T.J. Kop. | Bryophyta, Bryales, Miniaceae | Spain, Cuenca, Valdemeca, Arroyo Chico, 26-8-2012, J.A. Rosselló | 1 | 6, 7 |
| *Plagiomnium undulatum* (Hedw.) T.J. Koponen | Bryophyta, Bryales, Miniaceae | Slovenia: Bohinj lake, 23-8-2012, J. Miravet | 1 | 6, 7 |
| *Platyhypnidium riparioides* (Hedw.) Dix. | Bryophyta, Hypnales, Brachytheciaceae | Spain, Cuenca, Valdemeca, 26-8-2012, J.A. Rosselló | 1 | 8, 10, 11, 20 |
| *Pogonatum urnigerum* (Hedw.) P. Beauv. | Bryophyta, Polytrichales, Polytrichaceae | Spain, Lleida, between Tabascan and Noarre, 23-7-2012, J.A. Rosselló | 1 | 7 |
| *Pohlia cruda* (Hedw.) Lindb. | Bryophyta, Bryales, Mniaceae | Spain, Cuenca, Valdemeca, Arroyo Chico, 26-8-2012, J.A. Rosselló | 1 | 8, 10, 11, 14, 16, 22 |
| *Polytrichastrum alpinum* (Hedw.) G.L. Sm. | Bryophyta, Polytrichales, Polytrichaceae | Spain, Cuenca, Valdemeca, 20-5-2012, J.A. Rosselló | 1 | 7, 14 |
| *Polytrichum commune* Hedw. | Bryophyta, Polytrichales, Polytrichaceae | Spain, Lleida, Vall Ferrera, Pla de Boet, 25-7-2012, J.A. Rosselló | 1 | 7, 14 |
| *Polytrichum piliferum* Hedw. | Bryophyta, Polytrichales, Polytrichaceae | Spain, Cuenca, Valdemeca, 20-5-2012, J.A. Rosselló | 1 | 7, 14, 21 |
| *Porella cordaeana* (Huebener) Moore | Marchantiophyta, Porellales, Porellaceae | Spain, Cuenca, Valdemeca, Arroyo Chico, 26-8-2012, J.A. Rosselló | 1 | 8 |
| *Porella platyphylla* (L.) Pfeiff. | Marchantiophyta, Porellales, Porellaceae | Spain, Lleida, Pico Orri, 25-7-2012, J.A. Rosselló | 1 | 8 |
| *Pseudoscleropodium purum* (Hedw.) M. Fleisch. | Bryophyta, Hypnales, Brachytheciaceae | Spain, Cuenca, Valdemeca, 20-5-2012, J.A. Rosselló | 1 | 7, 11 |
| *Pterigynandrum filiforme* Hedw. | Bryophyta, Hypnales, Pterigynandraceae | Spain, Cuenca, Valdemeca, Arroyo Chico, 26-8-2012, J.A. Rosselló | 1 | - |
| *Pterigynandrum filiforme* Hedw. | Bryophyta, Hypnales, Pterigynandraceae | Slovenia: Bohinj lake, 23-8-2012, J. Miravet | 1 | - |
| *Racomitrium heterostichum* (Hedw.) Brid. | Bryophyta, Grimmiales, Grimmiaceae | Spain, Cuenca, Valdemeca, Arroyo Chico, 26-8-2012, J.A. Rosselló | 1 | 13 |
| *Reboulia hemisphaerica* (L.) Raddi | Marchantiophyta, Marchantiales, Aytoniaceae | Spain, Lleida, between Benabé and Pla de Beret, 24-7-2012, J.A. Rosselló | 1 | 9, 18 |
| *Rhizomnium punctatum* (Hedw.) T.J. Kop. | Bryophyta, Bryales, Mniaceae | Spain, Lleida, Vall Ferrera, Pla de Boet, 25-7-2012, J.A. Rosselló | 1* | 7 |
| *Rhizomnium punctatum* (Hedw.) T.J. Kop. | Bryophyta, Bryales, Mniaceae | Spain, Lleida, between Benabé and Pla de Beret, 24-7-2012, J.A. Rosselló | 1 | 7 |
| *Rhizomnium punctatum* (Hedw.) T.J. Kop. | Bryophyta, Bryales, Mniaceae | Spain, Cuenca, Valdemeca, Arroyo Chico, 26-8-2012, J.A. Rosselló | 1 | 7 |
| *Rhizomnium punctatum* (Hedw.) T.J. Kop. | Bryophyta, Bryales, Mniaceae | Spain, Cuenca, Valdemeca, Arroyo Chico, 26-8-2012, J.A. Rosselló | 1 | 7 |
| *Rhytidiadelphus triquetrus* (Hedw.) Warnst. | Bryophyta, Hypnales, Hylocomiaceae | Spain, Lleida, between Tabascan and Noarre, 23-7-2012, J.A. Rosselló | 1 | 6 |
| *Riella cf. notarisii* (Mont.) Mont. | Marchantiophyta, Sphaerocarpales, Riellaceae | Balearic Islands, Minorca, Cap Negre, 19-2-2012, J.A. Rosselló | 1 | 9 |
| *Sanionia uncinata* (Hedw.) Loeske | Bryophyta, Hypnales, Amblistegiaceae | Spain, Cuenca, Valtablado, 25-8-2012, J.A. Rosselló | 1 | 10, 11, 12 |
| *Scapania nemorea* (L.) Grolle | Marchantiophyta, Jungermanniales, Scapaniaceae | Slovenia: Bohinj lake, 23-8-2012, J. Miravet | 1* | 9 |
| *Scapania undulata* (L.) Dumort. | Marchantiophyta, Jungermanniales, Scapaniaceae | Spain, Cuenca, Valdemeca, 20-5-2012, J.A. Rosselló | 1 | - |
| *Schistidium apocarpum* (Hedw.) Bruch & Schimp. | Bryophyta, Grimmiales, Grimmiaceae | Spain, Cuenca, Valdemeca, Arroyo Chico, 26-8-2012, J.A. Rosselló | 1* | 14 |
| *Schistidium strictum* (Turner) Marten. | Bryophyta, Grimmiales, Grimmiaceae | Spain, Cuenca, Valdemeca, Arroyo Chico, 26-8-2012, J.A. Rosselló | 1 | 13 |
| *Scorpidium cossonii* (Schimp.) Hedenäs | Bryophyta, Hypnales, Amblistegiaceae | Spain, Cuenca, Valtablado, 25-8-2012, J.A. Rosselló | 1 | 11 |
| *Scorpiurium circinatum* (Brid.) M. Fleisch. & Loeske | Bryophyta ,Hypnales, Brachytheciaceae | Balearic Islands, Minorca, Pas den Revull, 20-2-2012, J.A. Rosselló | 1 | - |
| *Sphagnum* cf. *quinquefarium* (Braithw.) Warnst. | Bryophyta, Sphagnales, Sphagnaceae | Spain, Lleida, Vall Ferrera, 25-7-2012, J.A. Rosselló | 1 | 21 |
| *Sphagnum denticulatum* Brid. | Bryophyta, Sphagnales, Sphagnaceae | Spain, Cuenca, Valdemeca, 20-5-2012, J.A. Rosselló | 1* | 42 |
| *Sphagnum denticulatum* Brid. | Bryophyta, Sphagnales, Sphagnaceae | Spain, Lleida, Vall Ferrera, 25-7-2012, J.A. Rosselló | 1 | 42 |
| *Sphagnum denticulatum* Brid. | Bryophyta, Sphagnales, Sphagnaceae | Spain, Zamora, Sanabria, 6-8-2012, J. Miravet | 1 | 42 |
| *Sphagnum subnitens* Russow & Warnst. | Bryophyta, Sphagnales, Sphagnaceae | Spain, Cuenca, Valdemeca, 20-5-2012, J.A. Rosselló | 1 | - |
| *Sphagnum teres* (Schimp.) Ångstr. | Bryophyta, Sphagnales, Sphagnaceae | Spain, Lleida, between Tabascan and Noarre, 23-7-2012, J.A. Rosselló | 1* | 23, 24 |
| *Syntrichia ruralis* (Hedw.) F. Weber & D. Mohr var. *ruralis* | Bryophyta, Pottiales, Pottiaceae | Spain, Cuenca, Valdemeca, 20-5-2012, J.A. Rosselló | 1 | 12, 16 |
| *Syntrichia ruralis* (Hedw.) F. Weber & D. Mohr var. *ruralis* | Bryophyta, Pottiales, Pottiaceae | Spain, Cuenca, Valdemeca, Arroyo Chico, 26-8-2012, J.A. Rosselló | 1 | 12, 26 |
| *Targionia loorberiana* K. Müll. | Marchantiophyta, Marchantiales, Targioniaceae | Balearic Islands, Minorca, Pas den Revull, 20-2-2012, J.A. Rosselló | 1 | 27 |
| *Tetraphis pellucida* Hedw. | Bryophyta. Tetraphidales, Tetraphidaceae | Spain, Lleida, between Benabé and Pla de Beret, 24-7-2012, J.A. Rosselló | 1 | 8 |
| *Thamnobryum alopecurum* (Hedw.) Gangulee | Bryophyta, Hypnales, Neckeraceae | Slovenia: Bohinj lake, 23-8-2012, J. Miravet | 1 | 11 |
| *Thuidium delicatulum* (Hedw.) Schimp. | Bryophyta, Hypnales, Thuidiaceae | Slovenia: Bohinj lake, 23-8-2012, J. Miravet | 1 | 11 |
| *Thuidium tamariscinum* (Hedw.) Schimp. | Bryophyta, Hypnales, Thuidiaceae | Spain, Lleida, between Benabé and Pla de Beret, 24-7-2012, J.A. Rosselló | 1 | 11 |
| *Timmia austriaca* Hedw. | Bryophyta, Timmiales, Timmiaceae | Spain, Lleida, between Benabé and Pla de Beret, 24-7-2012, J.A. Rosselló | 1 | 8, 16 |
| *Tortella tortuosa* (Hedw.) Limpr. | Bryophyta, Pottiales, Pottiaceae | Slovenia: Bohinj lake, 23-8-2012, J. Miravet | 1 | 13 |
| *Ulota crispa* (Hedw.) Brid. | Bryophyta, Orthotrichales, Orthotrichaceae | Slovenia: Bohinj lake, 23-8-2012, J. Miravet | 1 | 11 |

*: Accessions not studied for the estimation of the rRNA gene copy number due to poor DNA quality.
